# Sex differences in pulmonary vascular control: focus on the nitric oxide pathway

**DOI:** 10.14814/phy2.13200

**Published:** 2017-06-08

**Authors:** Daphne P. M. de Wijs‐Meijler, A. H. Jan Danser, Irwin K. M. Reiss, Dirk J. Duncker, Daphne Merkus

**Affiliations:** ^1^ Division of Experimental CardiologyDepartment of CardiologyErasmus MCUniversity Medical Center RotterdamRotterdamThe Netherlands; ^2^ Division of NeonatologyDepartment of PediatricsSophia Children's HospitalErasmus MCUniversity Medical Center RotterdamRotterdamThe Netherlands; ^3^ Division of PharmacologyDepartment of Internal MedicineErasmus MCUniversity Medical Center RotterdamRotterdamThe Netherlands

**Keywords:** Exercise, nitric oxide, phosphodiesterase‐5, pulmonary vasculature, sex differences

## Abstract

Although the incidence of pulmonary hypertension is higher in females, the severity and prognosis of pulmonary vascular disease in both neonates and adults have been shown to be worse in male subjects. Studies of sex differences in pulmonary hypertension have mainly focused on the role of sex hormones. However, the contribution of sex differences in terms of vascular signaling pathways regulating pulmonary vascular function remains incompletely understood. Consequently, we investigated pulmonary vascular function of male and female swine in vivo, both at rest and during exercise, and in isolated small pulmonary arteries in vitro, with a particular focus on the NO‐cGMP‐PDE5 pathway. Pulmonary hemodynamics at rest and during exercise were virtually identical in male and female swine. Moreover, NO synthase inhibition resulted in a similar degree of pulmonary vasoconstriction in male and female swine. However, NO synthase inhibition blunted bradykinin‐induced vasodilation in pulmonary small arteries to a greater extent in male than in female swine. PDE5 inhibition resulted in a similar degree of vasodilation in male and female swine at rest, while during exercise there was a trend towards a larger effect in male swine. In small pulmonary arteries, PDE5 inhibition failed to augment bradykinin‐induced vasodilation in either sex. Finally, in the presence of NO synthase inhibition, the pulmonary vasodilator effect of PDE5 inhibition was significantly larger in female swine both in vivo and in vitro. In conclusion, the present study demonstrated significant sex differences in the regulation of pulmonary vascular tone, which may contribute to understanding sex differences in incidence, treatment response, and prognosis of pulmonary vascular disease.

## Introduction

Endothelial function is a key factor in vascular development as well as in maintenance of vascular structure and function throughout life. In the pulmonary vasculature, a healthy endothelium is essential for the transition from intrauterine to extrauterine life after birth, and endothelial dysfunction is an important factor in neonatal pulmonary vascular diseases such as bronchopulmonary dysplasia and neonatal pulmonary hypertension. Also later in life, endothelial dysfunction plays a critical role in the pathogenesis of adult pulmonary vascular disease, including pulmonary hypertension (PH). The pathogenesis of PH encompasses a combination of endothelial dysfunction, vasoconstriction, inflammation, structural remodeling of the pulmonary vasculature with formation of plexiform lesion and a high incidence of in situ thrombosis (Runo and Loyd 2003; Traiger 2007; Townsend et al. 2012; Montani et al. 2013).

Although the incidence of PH is estimated to be 2‐to 10‐fold higher in females than in males (Humbert et al. 2006; Badesch et al. 2010), the severity and prognosis of pulmonary vascular disease in both neonates and adults have been shown to be worse in male as compared to female subjects (Benza et al. 2010; Humbert et al. 2010). However, the mechanisms behind these sex differences are not completely understood. To date, research investigating sex differences in development and progression of pulmonary hypertension focused on the role of sex hormones, particularly female reproductive hormones. Although, sex hormones are thought to play an important role in the pathophysiology of pulmonary hypertension, it remains unclear whether estrogens and other sex hormones have a protective or detrimental effect (Chambliss and Shaul 2002; Smith et al. 2006; Tofovic 2010; Austin et al. 2013; Lahm et al. 2014; Martin and Pabelick 2014). Moreover, protective effects of estrogen are unlikely to explain all the sex differences in neonatal PH, at a time prior to full development of sex‐hormonal systems.

It is well known that the nitric oxide (NO) pathway plays an important role in the pathogenesis of pulmonary hypertension. In patients with pulmonary hypertension, NO deficiency contributes to the increased pulmonary vascular tone and vascular remodeling (Runo and Loyd 2003; Zhang et al. 2016). Although estrogen administration enhances eNOS activity in rat pulmonary vessels (Gonzales et al. 2001), the contribution of intrinsic sex‐related differences in the NO‐pathway to regulation of pulmonary vascular function remains incompletely understood. Consequently, the aim of the present study is to determine whether sex influences pulmonary vascular function through alterations in the NO pathway even in healthy conditions. For this purpose, we investigated the pulmonary vascular function in chronically instrumented male and female swine at rest and during treadmill exercise. We first compared the pulmonary vasodilator response to exercise. Subsequently, we investigated sex differences in the response of pulmonary small arteries to different vasoactive agents, that modulate the NO pathway, in vivo and in vitro.

## Materials and Methods

### In vivo animal experiments

Studies were performed in accordance with the “Guiding Principles in the Care and Use of Laboratory Animals” as approved by the Council of the American Physiological Society, and with approval of the Animal Care Committee of the Erasmus MC Rotterdam. Fifty‐nine crossbred Landrace x Yorkshire swine of 2–3 months old (31 males, 28 females) entered the study. Daily adaptation of animals to laboratory conditions started 1 week before surgery and continued during the first week after surgery.

### Surgical procedures

Swine were sedated with ketamine (20–30 mg kg^−1^ i.m.) and midazolam (1 mg kg^−1^ i.m.), anesthetized with thiopental (10–15 mg kg^−1^ i.v.), intubated, and ventilated with a mixture of O_2_ and N_2_ (1:2) to which 0.2–1% (v/v) isoflurane was added. Anesthesia was maintained with midazolam (2 mg kg^−1^ + 1 mg kg^−1^ h^−1^ i.v.) and fentanyl (10 *μ*g kg^−1^ h^−1^ i.v.), and the depth of anesthesia was checked regularly using a pain stimulus (toe‐pinch). Swine were instrumented under sterile conditions as previously described (De Wijs‐Meijler et al. 2016). Briefly, the chest was opened via the fourth left intercostal space and fluid‐filled polyvinylchloride catheters were inserted into the aortic arch, pulmonary artery and left atrium for pressure measurements. Furthermore, these catheters were used for blood sampling to determine the pO_2_, pCO_2_, pH, O_2_ saturation (sO_2_), and hemoglobin concentration (ABL 820, Radiometer) as well as for the infusion of drugs. A flow probe (14–16 mm, Skalar/Transonic) was positioned around the ascending aorta for measurement of cardiac output. Catheters and electrical wires were tunneled subcutaneously to the back and the chest was closed in layers. Animals were allowed to recover, receiving analgesia (0.3 mg buprenorphine i.m.) for 2 days and antibiotic prophylaxis (25 mg kg^−1^ amoxicillin and 5 mg kg^−1^ gentamicin i.v.) for 5 days. The catheters were flushed daily with heparinized saline (1000–5000 IE mL^−1^). After completing all experimental protocols, animals were killed by an intravenous overdose of pentobarbitone sodium.

### Experimental protocols

Studies were performed 1–3 weeks after surgery with swine exercising on a motor‐driven treadmill. Fluid‐filled pressure transducers were positioned on the back of the animals and calibrated at mid‐chest level. With swine (male, *n *=* *31 and female, *n *=* *28) resting on the treadmill, resting hemodynamic measurements, consisting of heart rate, cardiac output, mean aortic pressure (MAP), mean pulmonary artery pressure (PAP), and mean left atrial pressure (LAP), were obtained. Rectal temperature was measured, and arterial and mixed venous blood samples were collected. Subsequently, a five‐stage treadmill exercise protocol was started (1–5 km h^−1^); each exercise stage lasted 3 min. Hemodynamic variables were continuously recorded and blood samples were collected during the last 45 sec of each exercise stage, at a time when hemodynamics had reached a steady state (De Wijs‐Meijler et al. 2016). After completing the exercise protocol, animals were allowed to rest on the treadmill. After 90 min of rest, three different protocols were performed in a subset of swine, on different days and in random order (see below). The number of swine in each protocol, as well as overlap between protocols, is shown in Table 1. Excellent reproducibility of consecutive exercise trials has been reported previously (Duncker et al. 1998a, 2001).

**Table 1:** Schematic representation of the overlap of swine used in the different protocols

	LNNA	EMD360527	LNNA/EMD360527	Total
LNNA	**24M/19F**	5M/7F	5M/5F	
EMD360527	—	**7M/12F**	5M/4F	
LNNA/EMD360527	—	—	**5M/5F**	
Total				**31M/28F**

Bold values represent total number of animals per protocol.

### Effects of sex on the response to NO synthase inhibition during treadmill exercise

Ninety minutes after swine had undergone a control exercise trial (as described above) the NO synthase inhibitor N^*ω*^‐nitro‐L‐Arginine (LNNA, Sigma) was administered at a dose of 20 mg kg^−1^ i.v. in 24 male and 19 female swine. Ten minutes after completion of the infusion, resting measurements were obtained and the 5‐stage exercise protocol was repeated (Houweling et al. 2005; Merkus et al. 2006).

### Effects of sex on the response to PDE5 inhibition during treadmill exercise

Ninety minutes after swine had undergone a control exercise trial (as described above) the phosphodiesterase‐5 inhibitor EMD360527 (a gift from Merck, Darmstadt, Germany) was infused continuously in a dose of 300 *μ*g kg^−1 ^min^−1^ i.v. in 7 male and 12 female swine. Ten minutes after starting the infusion, resting measurements were obtained and the 5‐stage exercise protocol was repeated (Houweling et al. 2012; Zhou et al. 2014).

### Effects of sex on the response to PDE5 inhibition in the presence of NO synthase inhibition

Ninety minutes after swine had undergone a control exercise trial (as described above) the NO synthase inhibitor LNNA was administered in a dose of 20 mg kg^−1^ i.v. in five male and five female swine. Ten minutes after completion of the infusion, resting measurements were obtained and the 5‐stage exercise protocol was repeated. Ninety minutes later, animals received the phosphodiesterase‐5 inhibitor EMD360527 (300 *μ*g kg^−1 ^min^−1^ i.v.). Ten minutes after starting the infusion, resting measurements were obtained and swine underwent a third exercise trial (Houweling et al. 2005, 2012; Zhou et al. 2014).

### Blood gas measurements

Blood samples were maintained in iced syringes until the conclusion of each exercise trial. Measurements of paO_2_ (mmHg), paCO_2_ (mmHg), and pH, sO_2_ and hemoglobin (g/100 mL) were then immediately performed with a blood gas analyzer (ABL 820, Radiometer), and corrected for body temperature. Blood O_2_ content (*μ*mol/mL) was computed as follows: (Hb × 0.621 × sO_2_) + (0.00131 × pO_2_). Body O_2_ consumption (BVO_2_) was calculated as the product of cardiac output and the difference in O_2_ content between arterial and mixed venous blood.

### Data analysis and statistical analysis

Digital recording and off‐line analysis of hemodynamics have been described previously. (Duncker et al. 1998a, 2001). Pulmonary vascular conductance (PVC) was defined as cardiac output divided by mean PAP minus mean LAP. Systemic vascular conductance (SVC) was calculated as the ratio of cardiac output and mean arterial pressure. To accommodate for the varying weights between animals and groups, cardiac output, PVC, SVC, and BVO_2_ were indexed to body weight (Zhou et al. 2014).

Statistical analysis was performed using SPSS version 21.0 (IBM, Armonk, NY). Statistical significance was accepted at *P *≤* *0.05. Data are presented as mean ± SEM.

To test for the effects of sex and drug intervention on the relation between BVO_2_ and PVC, regression analysis was performed with sex, drug treatment, and BVO_2_, as well as their interactions as independent variables and animal as case label (SPSS version 21.0, IBM). Statistical analysis of the effect of drug intervention (vs. control) on PVC at rest and at maximal exercise was performed using unpaired *t*‐test to compare data from male and female swine (SPSS version 21.0, IBM).

### In vitro myograph experiments

Swine lungs (male, *n *=* *14 and female, *n *=* *13) were obtained at a local slaughterhouse. Pulmonary small arteries (diameter ≈ 300 *μ*m) were dissected out from the lower lung lobe and stored overnight in cold, oxygenated Krebs bicarbonate solution of the following composition (in mmol/L): NaCl 118, KCl 4.7, CaCl_2_ 2.5, MgSO_4_ 1.2, KH_2_PO_4_ 1.2, NaHCO_3_ 25, and glucose 8.3; pH 7.4. The next day, pulmonary small arteries were cut into segments of ~2 mm length and mounted in microvascular myographs (Danish MyoTechnology) with separate 6 mL organ baths containing Krebs bicarbonate solution aerated with 95% O_2_ and 5% CO2, and maintained at 37°C. Changes in contractile force were recorded with a Harvard isometric transducer. Following a 30 min stabilization period, the internal diameter was set to a tension equivalent to 0.9 times the estimated diameter at 20 mmHg effective transmural pressure. Vessels were then exposed to 30 mmol/L KCl twice. Endothelial integrity of pulmonary arteries was verified by observing dilation to 10 nmol/L substance P after precontraction with 100 nmol/L of the stable thromboxane A_2_ analog pyridoxalphosphate‐6‐azophenyl‐2′4′‐disulfonic acid (U46619). Vessels were then subjected to 100 mmol/L KCl to determine the maximal vascular contraction. Thereafter, pulmonary arteries were allowed to equilibrate in fresh Krebs solution for 30 min before initiating different experimental protocols (Zhou et al. 2014).

### Experimental protocols

After 30 min of equilibration in fresh Krebs, pulmonary small arteries were precontracted with 100 nmol/L U46619 before starting with one of four different experimental protocols (Zhou et al. 2014). Only one protocol was executed per vessel and within one protocol, each vessel was obtained from a different animal.

Pulmonary small arteries of male and female swine were subjected to the endothelium‐dependent vasodilator bradykinin (BK) in incremental concentrations ranging from 10^−10^ to 10^−7^ mol/L in the absence (15 male, 16 female) or presence of NO synthase inhibition with *N* ^*ω*^‐Nitro‐L‐arginine methyl ester hydrochloride (L‐NAME 10^−4^ mol/L, 11 male, 12 female), PDE5 inhibition with 10^−8^ mol/L sildenafil (8 male, 10 female) or combined NO synthase‐ and PDE5 inhibition (8 male, 10 female).

### Data analysis and statistical analysis

Vascular relaxation response to BK was expressed as percentage of contraction to U46619.

Statistical analysis was performed using SPSS version 21.0 (IBM) and Prism version 5.0 (Graphpad Software, Inc., La Jolla, CA). Statistical significance was accepted at *P *≤* *0.05. Data are presented as mean ± SEM.

The maximal relaxation (*E*_max_) and half maximal effective concentration (EC50) in each experiment was calculated using the GraphPad Prism version 5 for Windows (Graphpad Software, San Diego California, CA). Statistical analysis of maximal relaxation and EC50 to bradykinin was performed using two‐way (sex and drug intervention) analysis of variance (ANOVA) for repeated measures using SPSS version 21.0 (IBM). Statistical analysis of the effect of drug intervention (vs. control) at baseline and at bradykinin‐induced maximal relaxation was performed using unpaired *t*‐test to compare data from male and female swine (SPSS version 21.0, IBM).

### Quantitative real‐time PCR analysis

For detection of eNOS, soluble guanylyl cyclase (sGC) and PDE5A mRNA, pulmonary small arteries (diameter ≈ 300 *μ*m) were isolated from the same lungs as used in the myograph experiments, and snap frozen in liquid nitrogen. Small pieces of the frozen arteries (<30 mg) were homogenized by adding RLT lysisbuffer (Qiagen) using a homogenizer. After a prot K treatment at 55°C for 10 min, total RNA was isolated using RNeasy Fibrous Tissue Mini Kit (Qiagen). RNA was eluted in water and stored at −80°C. The concentration was determined by using a nanodrop and RNA integrity was confirmed by Bioanalyzer. cDNA was synthesized from 100 ng of total RNA with SensiFAST cDNA Synthesis Kit (Bioline). Quantitative real‐time PCR (CFX‐96, Bio‐Rad) was performed with SensiFAST SYBR & Fluorescein Kit (Bioline). Target gene mRNA levels were normalized against *β*‐actin, glyceraldehyde‐3‐phosphate dehydrogenase (GADPH), and Cyclophilin using the CFX manager software (Bio‐Rad). Primer sequences are shown in Table 2.

**Table 2:** Primer information

	Sequence	
Genes	Forward	Reverse
GAPDH	5′‐GCTCATTTCCTCGTACGACAAT‐3′	5′‐GAGGGCCTCTCTCCTCCTCGC‐3′
Actin	5′‐TCCCTGGAGAAGAGCTACGA‐3′	5′‐AGCACCGTGTTGGCGTAGAG‐3′
Cyclophilin	5′‐AGACAGCAGAAAACTTCCGTG‐3′	5′‐AAGATGCCAGGACCCGTATG‐3′
cGC	5′‐AATGGTACCCAGGAGTCACGC‐3′	5′‐ACGAACCAGGGAGAAGACAGA‐3′
eNOS	5′‐ GGACACACGGCTAGAAGAGC‐3′	5′‐TCCGTTTGGGGCTGAAGATG‐3′
PDE5A	5′‐GCCACTCAATCATGGAGCATC‐3′	5′‐GGAGAGGCCACTGAGAATCTG‐3′

## Results

### Effect of sex on hemodynamics during treadmill exercise

**Figure 1:**
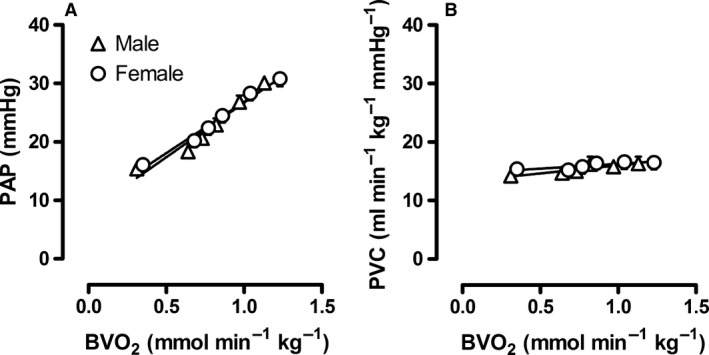
Changes in pulmonary hemodynamics during progressive levels of exercise in male and female swine. Relation between body oxygen consumption (BVO_2_) and (A) mean pulmonary arterial pressure (PAP) and (B) pulmonary vascular conductance (PVC). Values are mean ± SEM. There were no significant differences between male versus female swine.

Exercise up to 5 km h^−1^ produced a significant increase in cardiac output, while the mean aortic pressure was minimally affected (Table 3). (Fig. 1B). Exercise resulted in a twofold increase in pulmonary artery pressure (Fig. 1A), as a consequence of the increase in cardiac output, which caused an increase in the pressure drop across the pulmonary vasculature, in combination with the increase in left atrial pressure (Table 3). Due to the small vasodilator capacity of the lung vasculature PVC increased only slightly during exercise in both male (16 ± 7%) and female (9 ± 6%) swine (Fig. 1B). The hemodynamic responses during exercise were similar in both sexes (Table 3, Fig. 1).

**Table 3:** Hemodynamics in male and female swine

		Male		Female	
		Rest	Maximum exercise	Rest	Maximum exercise
HR (beats min^−1^)	Control	136 ± 3	249 ± 4	138 ± 5	254 ± 5
	Control	136 ± 4	251 ± 4	138 ± 5	252 ± 6
	LNNA	112 ± 3^a^	221 ± 5^a^	119 ± 3^a^	223 ± 6^a^
	Control	139 ± 5	262 ± 12	137 ± 8	255 ± 6
	EMD360527	147 ± 4	274 ± 12^a^	164 ± 6^a^^,^ c	260 ± 6
	Control	131 ± 5	256 ± 10	135 ± 7	250 ± 10
	LNNA	108 ± 3^a^	231 ± 12^a^	116 ± 6^a^	228 ± 9^a^
	LNNA + EMD360527	115 ± 7^a^	237 ± 13^a^	129 ± 10^a^	236 ± 8^a^
MAP (mmHg)	Control	86 ± 2	88 ± 2	86 ± 2	90 ± 2
	Control	86 ± 2	89 ± 2	88 ± 2	90 ± 2
	LNNA	117 ± 2^a^	116 ± 2^a^	117 ± 2^a^	121 ± 2^a^
	Control	84 ± 3	85 ± 3	84 ± 3	92 ± 4
	EMD360527	74 ± 4^a^	77 ± 3^a^	78 ± 3	83 ± 4^a^
	Control	76 ± 3	85 ± 2	88 ± 5	90 ± 4
	LNNA	111 ± 5^a^	111 ± 3^a^	112 ± 2^a^	119 ± 4^a^
	LNNA + EMD360527	106 ± 7^a^	104 ± 3^a^^,^ b	104 ± 7	107 ± 6^a^^,^ b
PAP (mmHg)	Control	16 ± 1	30 ± 1	16 ± 1	31 ± 1
	Control	15 ± 1	30 ± 1	17 ± 1	31 ± 1
	LNNA	23 ± 1^a^	40 ± 1^a^	26 ± 2^a^	44 ± 2^a^
	Control	18 ± 1	31 ± 1	15 ± 1	34 ± 2
	EMD360527	14 ± 1^a^	26 ± 3^a^	13 ± 1^a^	28 ± 2^a^
	Control	17 ± 2	33 ± 2	18 ± 1	31 ± 2
	LNNA	25 ± 3^a^	41 ± 3^a^	24 ± 3	44 ± 4^a^
	LNNA + EMD360527	18 ± 4^b^	32 ± 3^b^	14 ± 1^b^	33 ± 2^b^
LAP (mmHg)	Control	3 ± 1	10 ± 1	3 ± 1	9 ± 1
	Control	4 ± 1	10 ± 1	4 ± 1	11 ± 1
	LNNA	5 ± 1	10 ± 1	6 ± 1	11 ± 1
	Control	6 ± 1	12 ± 1	1 ± 1^a^	9 ± 2
	EMD360527	5 ± 1	11 ± 2	1 ± 1	10 ± 2
	Control	6 ± 1	12 ± 1	3 ± 1	9 ± 1
	LNNA	8 ± 1	12 ± 1	4 ± 2	9 ± 1
	LNNA + EMD360527	8 ± 3	14 ± 2	3 ± 2	10 ± 1
CI (l min^−^ kg^−^)	Control	0.17 ± 0.01	0.30 ± 0.01	0.19 ± 0.01	0.32 ± 0.01
	Control	0.18 ± 0.01	0.32 ± 0.01	0.19 ± 0.01	0.31 ± 0.01
	LNNA	0.14 ± 0.01^a^	0.27 ± 0.01^a^	0.15 ± 0.01^a^	0.27 ± 0.01^a^
	Control	0.21 ± 0.01	0.36 ± 0.01	0.19 ± 0.01	0.33 ± 0.02
	EMD360527	0.23 ± 0.01	0.38 ± 0.01^a^	0.21 ± 0.01	0.35 ± 0.02^a^
	Control	0.19 ± 0.01	0.34 ± 0.01	0.21 ± 0.01	0.37 ± 0.01^c^
	LNNA	0.15 ± 0.01^a^	0.28 ± 0.02^a^	0.18 ± 0.01^a^	0.32 ± 0.02^a^
	LNNA + EMD360527	0.17 ± 0.01	0.31 ± 0.01^a^^,^ b	0.21 ± 0.01^b^^,^ c	0.37 ± 0.01^b^^,^ c
PVCi (ml min^−1^ kg^−1^ mmHg^−1^)	Control	14 ± 1	16 ± 1	15 ± 1	17 ± 1
	Control	15 ± 1	18 ± 1	15 ± 1	16 ± 1
	LNNA	9 ± 1^a^	10 ± 1^a^	8 ± 1^a^	9 ± 1^a^
	Control	18 ± 1	20 ± 3	13 ± 1^c^	15 ± 2
	EMD360527	25 ± 2^a^	30 ± 4^a^	19 ± 2^a^	20 ± 3^a^^,^ c
	Control	15 ± 1	16 ± 2	15 ± 1	17 ± 2
	LNNA	8 ± 1^a^	10 ± 2^a^	9 ± 1^a^	10 ± 2^a^
	LNNA + EMD360527	14 ± 2^b^	16 ± 2^b^	22 ± 4^b^	17 ± 2^b^

Values are means ± SEM; *n* = 31 male and 28 female swine in the control group, *n* = 24 male and 19 female swine in the control/LNNA group, *n* = 7 male and 12 female swine in the control/EMD360527 group, and *n* = 5 male and 5 female swine in the control/LNNA/EMD360527 group. Maximum exercise is 5 km h^−1^.

HR, heart rate; MAP, mean arterial pressure; PAP, pulmonary artery pressure; LAP, left atrium pressure; CI, cardiac index; PVCi, pulmonary vascular conductance indexed for bodyweight.

a *P* ≤ 0.05 versus the corresponding control.

b *P* ≤ 0.05 LNNA + EMD360527 versus LNNA.

c *P* ≤ 0.05 female versus male swine at corresponding treadmill speed.

### Effect of sex on the response to bradykinin

**Figure 2:**
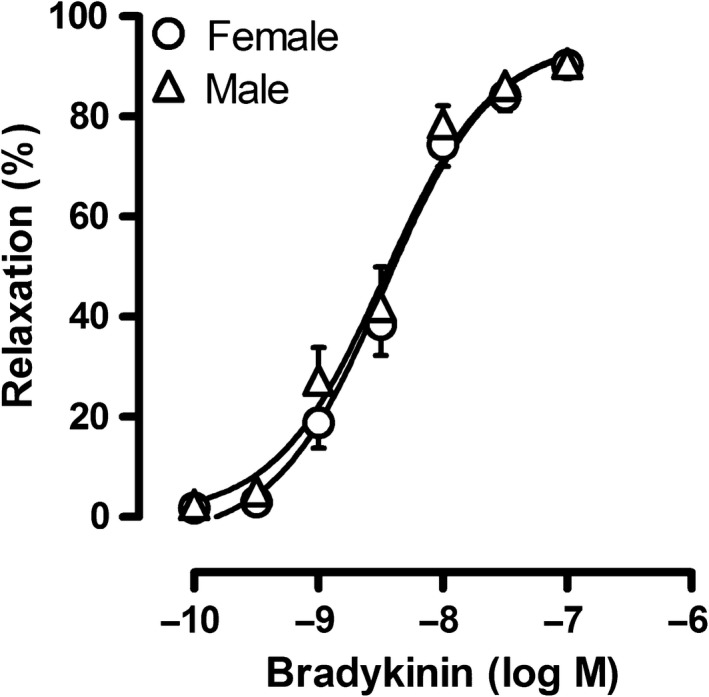
Concentration‐response to bradykinin in pulmonary small arteries from male and female swine precontracted with U46619 (100 nmol/L). Values are mean ± SEM. There were no significant differences between male versus female swine.

Cumulative concentrations of bradykinin produced a concentration‐dependent vasodilation up to 100% in precontracted isolated porcine pulmonary small arteries from either male or female sex. This vasodilator response to bradykinin was similar in pulmonary small arteries from male as compared to female swine, both in terms of *R*_max_ and EC50 (Fig. 2).

### Effect of sex on the response to NO synthase inhibition

**Figure 3:**
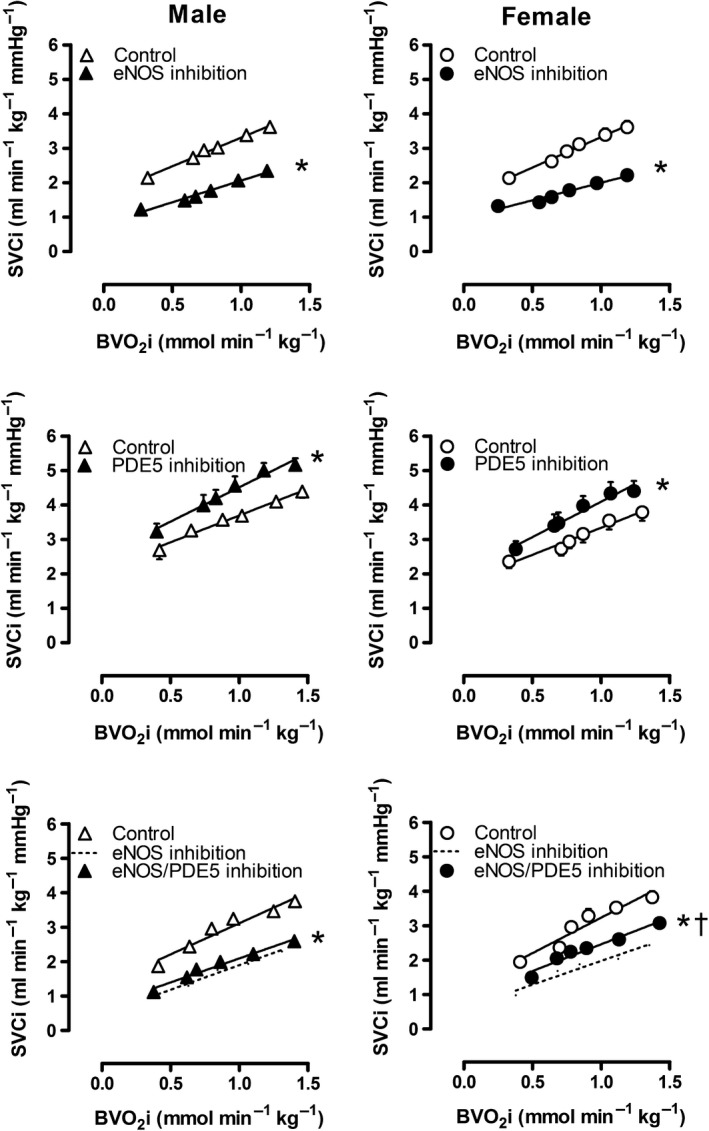
Effect of NO synthase and/or PDE5 on systemic vascular conductance in vivo at rest and during exercise. Values are mean ± SEM. *P ≤ 0.05 versus corresponding control; ^†^ *P* ≤ 0.05 female versus male swine. SVC, systemic vascular conductance; BVO_2_, body oxygen consumption.

**Figure 4:**
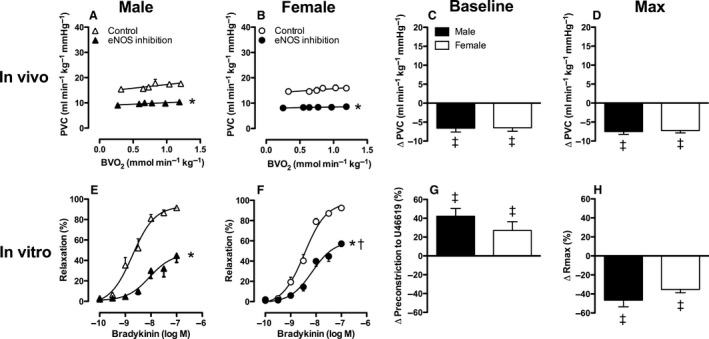
Effect of NO synthase on pulmonary vascular conductance in vivo at rest and during exercise (Panels A–D) and on bradykinin‐induced vasodilation in vitro (Panels E–H) in male and female swine. Panels C and G show the change at baseline and panels D and H show the change at maximum compared to control. Values are mean ± SEM. *P ≤ 0.05 versus corresponding control; ^†^ *P* ≤ 0.05 female versus male swine; ^‡^ *P* ≤ 0.05 versus no change in Pulmonary vascular conductance. (e.g., vs. zero). PVC, Pulmonary vascular conductance; Rmax, maximum relaxation.

Administration of the NO synthase inhibitor LNNA in vivo increased mean aortic pressure (Table 3) and decreased systemic vascular conductance (Fig. 3) to a similar extent in male and female swine, both at rest and during incremental levels of exercise. This increase in aortic pressure was accompanied by a, probably baroreceptor reflex mediated, decrease in heart rate, and CI (Table 3). Similar to the systemic hemodynamic response, PAP markedly increased after LNNA administration. This increase in PAP was the result of extensive pulmonary vasoconstriction, as evidenced by a marked decrease in PVC. However, neither the LNNA‐induced decrease in PVC at rest, nor the effect of LNNA on PVC during exercise was significantly different between male and female swine (Table 3, Fig. 4A–D).

In vitro, addition of the NO synthase inhibitor L‐NAME (10^−4^mol/L) significantly enhanced the precontraction to U46619 (Fig. 4G), and reduced the maximum bradykinin‐induced relaxation (Fig. 4E, F and H). The concentration‐response of pulmonary arteries from both male and female swine to bradykinin was shifted significantly to the right in the presence of L‐NAME (EC50: Male 1.9 nmol/L vs. 8.8 nmol/L, *P *= 0.01; Female 3.5 nmol/L vs. 7.0 nmol/L, *P *= 0.02). In contrast to the comparable effect of NO synthase inhibition in both sexes in vivo, the BK‐induced relaxation after L‐NAME administration was significantly smaller in male swine as compared to female swine (Fig. 4E, F). However, the mRNA expression levels of eNOS in small pulmonary arteries (*P *=* *0.84) were not statistically different between sexes.

### Effect of sex on the response to PDE5 inhibition

**Figure 5:**
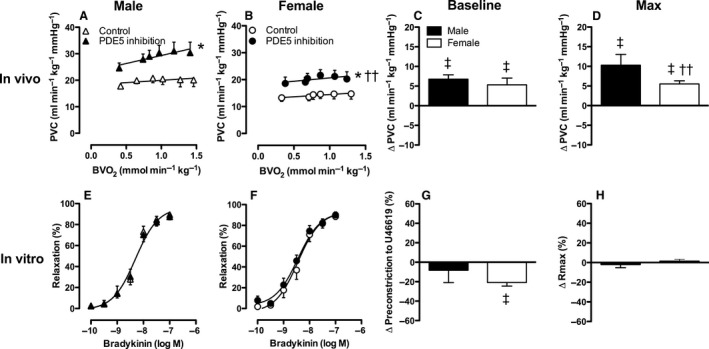
Effect of PDE5 on pulmonary vascular conductance in vivo at rest and during exercise (Panels A–D) and on bradykinin‐induced vasodilation in vitro (Panels E–H) in male and female swine. Panels C and G show the change at baseline and panels D and H show the change at maximum compared to control. Values are mean ± SEM. *P ≤ 0.05 versus corresponding control; ^†^ *P* ≤ 0.05 female versus male swine; ^††^ *P* ≤ 0.1 female versus male; ^‡^ *P* ≤ 0.05 versus no change in Pulmonary vascular conductance (e.g., vs. zero). PVC, Pulmonary vascular conductance; Rmax, maximum relaxation.

PDE5 inhibition resulted in a significant increase in systemic vascular conductance at rest (Fig. 3) that was accompanied by a, decrease in mean aortic pressure (Table 3). The effects of PDE5 inhibition on both SVC and MAP were sustained with graded treadmill exercise (Table 3). In the pulmonary circulation, PDE5 inhibition decreased pulmonary artery pressure significantly both at rest and during exercise. This decrease in PAP was the result of pulmonary vasodilation, as PDE5 inhibition increased PVC at rest and during exercise (Table 3, Fig. 5A–D), while left atrial pressure was unaffected and CO increased, particularly during exercise. However, neither the increase in PVC at rest produced by PDE5 inhibition, nor the effect of PDE5 inhibition during exercise were different in male as compared to female swine (Table 3, Fig. 5A–D), although a trend towards a significantly larger effect of PDE5 inhibition during exercise in male swine was found (*P *=* *0.077 for PDE5 inhibition x sex, Fig. 5A, B and D). These data suggest that endogenous PDE5 may have a more pronounced pulmonary vasoconstrictor role in male swine, particularly during exercise (Table 3, Fig. 5A, B and D).

Although PDE5 inhibition by sildenafil (10^−8^mol/L) tended to attenuate the precontraction to U46619 (Fig. 5G), it failed to augment the bradykinin‐induced vasodilation in small pulmonary arteries isolated from either male or female swine. Hence, no sex differences in response to PDE5 inhibition were found in vitro (Fig. 5E, F and H). Similarly, no significant differences were found in PDE5A mRNA expression levels (*P *=* *0.12).

### Effect of sex on the response to PDE5 inhibition in the presence of NO synthase inhibition

In the systemic circulation, PDE5 inhibition following NO synthase inhibition decreased mean aortic pressure only in female swine, at rest as well as during exercise. (Table 3). This decrease in mean aortic pressure was accompanied by a significant increase in CI and SVC (Table 3, Fig. 3). In male swine, the effect of PDE5‐inhibtion in the systemic vasculature following NO synthase inhibition only reached statistical significance during exercise. However, mean aortic pressure remained significantly higher as compared to control conditions in both male and female swine. In contrast, CI in the presence of combined NO synthase and PDE5 inhibition remained significantly lower as compared to control conditions in male swine only (Table 3).

**Figure 6:**
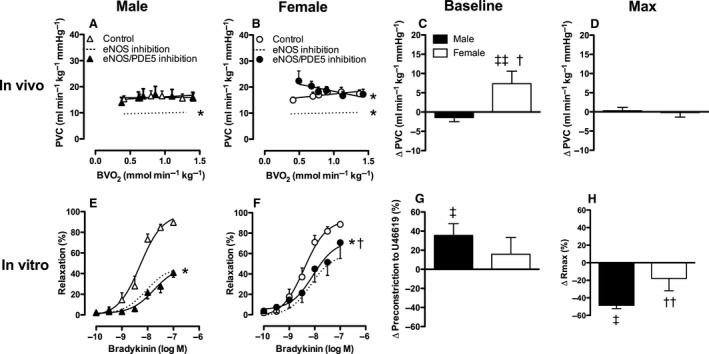
Effect of NO synthase and PDE5 on pulmonary vascular conductance in vivo at rest and during exercise (Panels A–D) and on bradykinin‐induced vasodilation in vitro (Panels E–H) in male and female swine. Panels C and G show the change at baseline and panels D and H show the change at maximum compared to control. Values are mean ± SEM. *P ≤ 0.05 versus corresponding control; ^†^ *P* ≤ 0.05 female versus male swine; ^††^ *P* ≤ 0.1 female versus male; ^‡^ *P* ≤ 0.05 versus no change in PVC (e.g., vs. zero); ^‡‡^ *P* ≤ 0.1 versus no change in PVC (e.g., vs. zero). PVC, Pulmonary vascular conductance; Rmax, maximum relaxation.

PDE5 inhibition, with EMD360527, following NO synthase inhibition, restored pulmonary artery pressure and PVC to control levels in both male and female swine (Table 3, Fig. 6A–D). In female swine, PVC at rest tended to be even higher as compared to control (*P *=* *0.08). The change in PVC in response to combined PDE5 ‐and NO synthase inhibition was significantly smaller in male as compared to female swine at rest (Fig. 6C). However, no significant sex differences in the PDE5 inhibition induced increase in PVC during exercise were observed (Table 3, Fig. 6A, B and D).

BK‐induced vasodilation was significantly blunted by combined NO synthase and PDE5 inhibition in pulmonary small arteries from male swine and to a lesser extent in female pulmonary small arteries (Fig. 6E, F and H). Furthermore, there was a significant rightward shift of the concentration‐response curve to bradykinin in the presence of L‐NAME and sildenafil compared to control, but this shift was identical in male and female swine.

Altogether, these data suggest that there is a pathway different from the NO pathway that results in activation of sGC or pGC to produce cGMP. Furthermore, both in vivo and in vitro data indicate that in the presence of NO synthase inhibition, PDE5 inhibition has a larger pulmonary vasodilator effect in female, as compared to male swine. Since the mRNA expression levels of sGC did not differ between male and female swine (*P *=* *0.20), this larger pulmonary vasodilator effect in female swine is unlikely to be explained by a higher presence of sGC.

## Discussion

This study compared the pulmonary vascular responses of healthy, young adolescent male and female swine both in vivo and in vitro to investigate whether sex differences are noted at an early age. The main findings of this study were that (1) pulmonary hemodynamics at rest and during incremental exercise did not differ between male and female swine. (2) NO synthase inhibition with LNNA resulted in pulmonary vasoconstriction to a similar extent in male and female swine at rest and during exercise. (3) NO synthase inhibition with L‐NAME also reduces BK‐induced vasodilation in isolated pulmonary small arteries; however this reduction was slightly larger in male than in female swine. (4) PDE5 inhibition with EMD360527 resulted in pulmonary vasodilation to a similar extent in male and female swine at rest and during exercise, but PDE5 inhibition with sildenafil failed to augment the bradykinin‐induced vasodilation in small pulmonary arteries isolated from either male or female swine. (5) In the presence of NO synthase inhibition, the pulmonary vasodilator effect of PDE5 inhibition was significantly larger in female swine both in vivo (at rest) and in vitro. The implications of the present findings are discussed below.

### Methodological considerations

#### Determination of PVC

PVC is defined as cardiac output divided by mean PAP minus mean pulmonary backpressure. Pulmonary backpressure can be measured either by pulmonary wedge pressure or by left atrial pressure (Merkus et al. 2008). In the clinical setting, pulmonary wedge pressure is routinely used to determine the pulmonary backpressure, because it can be easily obtained by the same catheter that is used to measure pulmonary artery pressure, thereby circumventing the need to insert a catheter in the left atrium. It is measured by inflating a balloon in a branch of the pulmonary artery to empty the vasculature distal to the balloon. The pressure distal to the balloon rapidly falls, and after several seconds, reaches a stable lower value that is very similar to the pulmonary backpressure. To prevent overestimation of wedge pressure, it is important to allow sufficient time for emptying the vasculature distal to the balloon (Merkus et al. 2008). Direct measurement of left atrial pressure, meanwhile, circumvents these concerns associated with wedge pressure measurement and allows continuous assessment of PVC. Importantly, left atrial pressure measurements (Duncker et al. 1998b; Stubenitsky et al. 1998; de Beer et al. 2010) correspond very well with wedge pressure measurements (Hopkins et al. 1999) at comparable levels of heart rate in swine at rest and during exercise, suggesting that either measurement provides a good measurement of pulmonary back pressure.

#### Selectivity of NO synthase inhibitors

Nitric oxide synthases (NOSs) are enzymes catalyzing the production of nitric oxide from L‐arginine. Two classes of NOSs exist; the constitutive isoforms endothelial NOS (eNOS) and neuronal NOS (nNOS), and the inducible isoform (iNOS). Both LNNA and L‐NAME demonstrate a 10‐ to 30‐fold selectivity for eNOS and nNOS over iNOS (Boer et al. 2000).

It has been previously shown that LNNA 20 mg kg^−1^ i.v. blunts the vasodilator response to ATP, but not to SNP, indicating that the effect of LNNA is specific to the endothelium‐dependent vasodilation (Duncker et al. 2000). The dose we used in the present study was based on a study in swine from our laboratory that has shown that higher doses (40 mg kg^−1^ i.v.) produced similar hemodynamic responses compared to 20 mg kg^−1^ (Duncker et al. 2000). LNNA in the concentration used in this study, however, blocks all three isoforms of NOS (Boer et al. 2000). Since iNOS blockade with aminoguanidine had no effect on basal pulmonary vascular tone at rest or during exercise in either healthy swine or in swine with myocardial infarction (Haitsma et al. 2002), it is unlikely that iNOS is involved in regulation of pulmonary vascular tone. However, since NO released from perivascular nerves has been shown to exert a vasodilator effect on the pulmonary vasculature (Toda et al. 2009), an effect of nNOS inhibition by LNNA on pulmonary vascular tone cannot be excluded in this study.

In our in vitro experiments, L‐NAME was used in a dose of 10^−4 ^mol/L. This dose of LNAME results in a complete blockade of eNOS (IC_50 _= 0.09 *μ*mol/L) and nNOS (IC_50 _= 0.05 *μ*mol/L) (Boer et al. 2000). Since pulmonary small arteries were dissected from the lung, thereby interrupting perivascular nerve signaling, it is unlikely that NO produced by nNOS played a role in the vasodilator response to bradykinin.

#### Selectivity of PDE5 inhibitors

Phosphodiesterases are enzymes responsible for the degradation of cAMP and cGMP in a wide variety of cell types. To date, at least 11 different families of PDEs have been identified, all with different kinetic properties, localization, and function. In vascular smooth muscle, PDE1 and particularly PDE5 are responsible for the degradation of the bulk of cGMP, while PDE3 is responsible for degradation of cAMP (Bender and Beavo 2006). PDE5 inactivates cGMP by hydrolyzing it to 5′ GMP. PDE inhibitor sildenafil demonstrates at least fivefold selectivity for PDE5 (IC_50_ = 0.0085 *μ*mol/L) compared with PDE6 (IC_50 _= 0.049 *μ*mol/L), 41‐fold selectivity for PDE1 (IC_50 _= 0.35 *μ*mol/L), 376‐fold selectivity for PDE4 (IC_50_ = 3.2 *μ*mol/L), 447‐fold selectivity for PDE10 (IC_50_ = 3.8 *μ*mol/L), and >1000‐fold selectivity for PDE2, PDE3, and PDE7 (IC_50_ > 10 *μ*mol/L) (Bischoff 2004). PDE inhibitor EMD‐360527 demonstrates at least 45‐fold selectivity for PDE5 (IC_50_ = 0.007 *μ*mol/L) compared with PDE6 (IC_50 _= 0.32 *μ*mol/L), 94‐fold selectivity for PDE1 (IC_50 _= 0.66 *μ*mol/L), 137‐fold selectivity for PDE10 (IC_50_ = 0.96 *μ*mol/L), and >1,400‐fold selectivity for PDE2, PDE3, PDE4, and PDE7 (IC_50_ > 10 *μ*mol/L) (Houweling et al. 2012). In this study, we used EMD360527 in a dose of 300 *μ*g kg^−1 ^min^−1^ i.v., resulting in plasma drug concentration of 15 *μ*mol/L (Merkus et al. 2013). Although inhibition of PDEs other than PDE5 may have occurred at this concentration, this is unlikely given our observation that EMD360527 has negligible effects on LV dP/d*t*max compared with the PDE3 inhibitor pimobendan that increases cAMP (Merkus et al. 2013).

### Experimental animals

In male swine, the production of sex hormones starts at approximately 3 months of age and sexual maturity is attained at the age of 6–7 months. Puberty in female swine usually takes place at an age of 5–7 months (Andersson et al. 1999). Animals entering the study for the in vivo exercise experiments were juvenile swine at an age of 2–3 month old; male swine had been neutered. The use of juvenile, neutered animals eliminates long‐term exposure to sex hormones, which potentially leads to lasting changes in pulmonary vascular function and structure in adult animals. Hence, the influence of sex hormones on the sex differences in pulmonary vascular control in our in vivo experiments was likely negligible. Pulmonary small arteries for the in vitro myograph experiments were isolated from lungs from 4‐ to 6‐month old swine, obtained at a local slaughterhouse; male swine were not neutered (prohibited by Dutch law since 2015). Since the production of sex hormones has already started at this age, it cannot be excluded that sex hormone exposure, especially in male swine that attained sexual maturity, influenced structure and function of the pulmonary vasculature. However, although an influence of sex hormones on pulmonary vascular structure cannot be excluded in the in vitro experiments, when isolated and mounted in Mulvany organ baths, the influence of circulating estrogens on eNOS activity in the pulmonary arteries is no longer present, and hence it is unlikely that sex hormones influence the outcome of the in vitro experiments.

### Sex differences in pulmonary vascular control

Sex differences in the NO pathway have been observed in the systemic vasculature. Thus, eNOS protein expression is higher in both skeletal muscles of female swine (Laughlin et al. 2003), and coronary and cerebral arteries of female rats (Knot et al. 1999; Arrick et al. 2016). Consistent with the higher eNOS protein expression in females, NO urine excretion was also higher in healthy women as compared to men (Forte et al. 1998). To the best of our knowledge, our study is the first to comprehensively investigate sex differences in the NO pathway in the healthy pulmonary vasculature.

Pulmonary hemodynamics at rest and during exercise were similar in male and female swine. The vasodilator response to bradykinin was also similar in pulmonary small arteries from male as compared with female swine, both in terms of *R*_max_ and EC50. In contrast to the above‐mentioned studies in the systemic vascular endothelial cells, we observed no differences in eNOS mRNA expression in pulmonary small arteries from sexually mature male as compared to female swine. Although it is possible that the translation of mRNA in protein differs between male and female subjects, the unchanged mRNA expression suggests that the influence of sex on eNOS expression depends on the vascular bed studied (systemic vs. pulmonary). These findings are consistent with the comparable pulmonary vasoconstriction produced by NO synthase inhibition both at rest and during exercise in vivo between male and female swine. In vitro, NO synthase inhibition reduced BK‐induced vasorelaxation in pulmonary small arteries from male swine to a greater extent as compared to female swine. Although this apparent discrepancy between in vivo and in vitro findings is not readily explained, several possible explanations could be forwarded. First, eNOS activation by circulating estrogen in the pulmonary circulation (Chambliss and Shaul 2002; Smith et al. 2006) is present in vivo but not in vitro. In the present study, the influence of estrogens is rather unlikely because the in vivo experiments were performed prior to puberty. Second, both eNOS and nNOS produce NO in the pulmonary vasculature in vivo, and thereby contribute to regulation of pulmonary vascular tone (Toda et al. 2009). Miller et al. (2005) showed that deletion of the eNOS gene has a greater impact on the pulmonary circulation of male than female mice. Together with the findings in the present study that LNNA (inhibition of both eNOS and nNOS) had a similar effect on pulmonary vascular tone in male and female swine, a higher eNOS/nNOS ratio in male as compared to female subjects is likely. This is consistent with our observation that in vitro, in the absence of a contribution of nNOS, NO synthase inhibition has a greater effect in male as compared to female pulmonary arteries. Third, receptor‐mediated eNOS activation is known to act through a different signaling pathway than shear stress‐mediated eNOS activation. Thus, receptor‐mediated activation of eNOS occurs through a calcium calmodulin‐dependent pathway (Motte et al. 1995), whereas shear stress activates eNOS through Akt‐mediated phosphorylation (Dimmeler et al. 1999), resulting in calcium‐independent activation of eNOS. Several studies have shown that estrogen rapidly activates eNOS via a phosphoinositide‐3 (PI‐3) kinase‐dependent pathway (Haynes et al. 2002; Stirone et al. 2005; Hohmann et al. 2016). Hence, it is possible that sex affects calcium handling of the endothelial cells. Finally, sex differences have been found in NO signaling, which occurred solely through cGMP‐PKG‐PDE5 in males, whereas an unidentified alternative vasodilator pathway was present in females (Chan et al. 2012; Wong et al. 2014). Consistent with these findings, we have previously shown that in the pulmonary vasculature, a significant part of the NO‐mediated vasodilator effect resulted from inhibition of endothelin‐signaling, although we did not investigate the effect of sex in that study (Houweling et al. 2006). Alternatively, endothelium‐derived hyperpolarizing factor (EDHF) pathways may play a pivotal role in governing vascular tone in the pulmonary vasculature of female, but not in male subjects, like it has been shown previously in the systemic circulation (Scotland et al. 2005; Chan et al. 2012; Pessoa et al. 2015). Future studies are required to investigate in more detail the mechanisms underlying the different responses in vitro and in vivo, in male versus female swine.

The vasodilator effect of PDE5 inhibition on the intact pulmonary vasculature in awake resting swine, was similar in male and female swine. During exercise, a trend towards an increased vasodilator effect of PDE5 inhibition during exercise in male as compared to female swine (*P *=* *0.077), was observed suggesting a more active endogenous PDE5 in male swine. This is consistent with recent post hoc analyses of the PHIRST and SUPER trials (Galie et al. 2005; Mathai et al. 2015), showing that PDE5 inhibition lead to a greater improvement of 6 min walking distance in male as compared to female patients with PAH. Interestingly, PDE5 inhibition had no significant effect on BK‐induced vasodilation of isolated pulmonary arteries from swine of either sex under baseline conditions, but blunted the LNAME‐mediated inhibition of BK vasodilation in pulmonary small arteries from female but not male swine. Similarly, in the presence of NO synthase inhibition, PDE5 inhibition produced a larger vasodilator effect in the intact pulmonary vasculature of female swine as compared to male swine at rest, while this difference was abrogated during exercise. These divergent responses to PDE5 inhibition during control conditions (larger in males during exercise) and during concomitant NO synthase inhibition (larger in females at rest and during BK), in male versus female swine are not readily explained. However, we have previously shown that the sensitivity of the pulmonary vasculature to cGMP is enhanced by NO synthase inhibition (Houweling et al. 2012). This study suggests that an increased sensitivity to cGMP produced by NO synthase inhibition is particularly pronounced in female swine, which might be related to different signaling pathways of NO in female versus male swine (Chan et al. 2012; Wong et al. 2014). The exact mechanisms underlying these divergent responses in male and female swine should be the subject of future studies.

### Conclusions and clinical implications

In conclusion, the present study demonstrated significant sex differences in the regulation of pulmonary vascular tone. Thus, NO synthase inhibition reduced BK‐induced vasorelaxation to a greater extent in male as compared to female pulmonary small arteries, which is consistent with observations that pulmonary vascular diseases is often more severe in men as compared to women. The increased vasodilator effect of PDE5 inhibition during exercise in male as compared to female swine reflects the sex‐specific heterogeneity in treatment response. Finally, concomitant NO synthase inhibition enhanced the vasodilator responses to PDE5 inhibition at rest and during BK‐induced vasodilation, but only in females, suggesting that loss of endothelial function may not interfere with (males) or even enhance (females) the pulmonary vasodilator responses to PDE5 in patients with pulmonary hypertension. Future studies are required to further investigate the mechanisms underlying these sex‐related differences in pulmonary vascular control mechanisms.

## Conflict of Interest

The authors do not have any potential or actual personal, political, or financial interest in the material, information, or techniques described in this paper.
